# Plasma interferon predicts pulmonary hypertension severity and outcome

**DOI:** 10.3389/fcvm.2025.1634764

**Published:** 2025-11-05

**Authors:** Zhanping Liang, Yun Li, Shuying Jia, Yuyan Liu, Jie Zhu, Jinling Li, Fei Li, Xiaohuan Xia

**Affiliations:** ^1^ Center for Translational Neurodegeneration and Regenerative Therapy, Shanghai Tenth People’s Hospital Affiliated to Tongji University, Shanghai, China; ^2^ Department of Cardio-Pulmonary Circulation, Shanghai Pulmonary Hospital, School of Medicine, Tongji University, Shanghai, China; ^3^ Department of Radiology, Shanghai Pulmonary Hospital, School of Medicine, Tongji University, Shanghai, China; ^4^ Center for Translational Neurodegeneration and Regenerative Therapy, Tongji Hospital Affiliated to Tongji University, Shanghai, China; ^5^ State Key Laboratory of Cardiology and Medical Innovation Center, Shanghai East Hospital, School of Medicine, Tongji University, Shanghai, China; ^6^ Shanghai Key Laboratory of Anesthesiology and Brain Functional Modulation, Shanghai Fourth People’s Hospital, School of Medicine, Tongji University, Shanghai, China; ^7^ Department of Anesthesiology, Tongji Hospital Affiliated to Tongji University, Shanghai, China; ^8^ Translational Research Institute of Brain and Brain-Like Intelligence, Shanghai Fourth People’s Hospital Affiliated to Tongji University School of Medicine, Shanghai, China; ^9^ Innovation Center of Medical Basic Research for Brain Aging and Associated Diseases, Ministry of Education, Tongji University, Shanghai, China

**Keywords:** pulmonary hypertension, inflammation, interferon, risk factor, prognosis

## Abstract

**Background:**

Pulmonary hypertension (PH) is a complex and progressive disease characterized by elevated pulmonary vascular resistance. Inflammation plays an important role in the pathogenesis of PH. Interferons (IFNs) are key immune cytokines that regulate cellular responses to various stimuli. This study aimed to investigate the association between IFN levels and the risk and prognosis of PH.

**Methods:**

A cohort of 875 PH patients and 182 matched controls were included in this study. Logistic regression models and receiver operating characteristic (ROC) curves were applied to evaluate the association between IFN levels and PH risk. Correlations between variables were assessed using Spearman's rank correlation coefficient (r ). Cox proportional hazards regression and Kaplan-Meier survival curves were used to assess the prognostic value of IFNs levels. The predictive performance of prognostic models was assessed using ROC analysis and decision curve analysis and a nomogram was constructed to estimate individual overall survival probabilities.

**Results:**

Both IFN-α and IFN-γ levels were significantly elevated in PH patients compared with controls (*P* < 0.05 for both), with IFN-α showing stronger predictive value across multiple PH subgroups (*P* < 0.05 for all), particularly in patients with Group 4 PH and idiopathic pulmonary arterial hypertension (IPAH). In Cox regression models, IFN-α was significantly associated with lower survival in PH patients (HR: 1.120, 95% CI: 1.001-1.253, *P* = 0.048). Kaplan-Meier analysis demonstrated that patients with IFN-α > 2.131 pg/mL had significantly lower 5-year cumulative survival rate of 60.1%, compared with 81.2% for those with IFN-α ≤ 2.131 pg/mL (Log Rank *P* < 0.001). A prognostic model combining IFN-α with traditional clinical markers, such as WHO-FC, 6-MWD, and NT-proBNP, improved predictive accuracy, with IFN-α contributing additional clinical net benefit in risk stratification.

**Conclusions:**

These findings suggest that plasma IFN-α may serve as a valuable biomarker for both predicting PH risk and assessing prognosis.

## Introduction

Pulmonary hypertension (PH) is a group of cardiopulmonary disorders characterized by elevated mean pulmonary artery pressure (mPAP > 20 mmHg) (1). Five major groups of PH are recognized: pulmonary arterial hypertension (PAH, Group 1, G1), PH associated with left-sided heart disease (Group 2, G2), PH related to chronic lung disease or hypoxia (Group 3, G3), PH resulting from chronic thromboembolic disease (Group 4, G4), and PH with unclear and/or multifactorial mechanisms. PAH, a major subset of PH, can be further classified into idiopathic pulmonary arterial hypertension (IPAH), connective tissue disease-associated PAH (CTD-PAH), and congenital heart disease-associated PAH (CHD-PAH) (2, 3). Despite their distinct etiologies, all forms of PH share common pathological features, including adverse vascular remodeling and right ventricular hypertrophy, which ultimately lead to right heart failure, the major cause of mortality in PH patients (4–6).

Inflammation has long been recognized as a key contributor to the pathogenesis of PH (7, 8). Elevated levels of various inflammatory mediators have been reported in both PH patients and experimental models (9–11). The intravascular infiltration of immune cells such as macrophages, B cells, and mast cells indicates immune dysregulation during PH progression (12, 13). Multiple inflammatory cytokines including interleukins (IL-1β, IL-6, and IL-10), tumor necrosis factor-α (TNF-α), and interferons (IFNs), are implicated in the initiation and progression of vascular remodeling (14–16). IFNs play critical roles in regulating immune cell activity, inflammatory signaling, and endothelial function (5). IFNs also regulate the transcription of numerous interferon-stimulated genes (ISGs), such as interferon regulatory factor 7 (IRF7) and interferon-inducible protein 44 (IFI44), which have been shown to modulate pulmonary vascular remodeling in animal models of PH (17, 18). Despite increasing evidence of IFNs upregulation in PH patients, the association between IFN levels and the risk or survival of PH patients remains unclear (12).

In this study, we sought to determine whether plasma IFN levels, particularly IFN-α, could serve as reliable predictors of disease risk and patient prognosis by examining the correlation between IFNs and clinical outcomes. Furthermore, we aimed to develop a IFNs-based prognostic model to improve the accuracy of PH risk stratification and survival prediction.

## Methods

### Study population

This observational study was conducted at Shanghai Pulmonary Hospital using data collected from January 2012 to April 2023. A total of 875 patients diagnosed with pulmonary hypertension (PH) and 182 age- and gender-matched controls were included. The diagnostic criteria for PH were defined as mean pulmonary artery pressure (mPAP) >20 mmHg, pulmonary vascular resistance (PVR) ≥3 Wood units, and pulmonary artery wedge pressure (PAWP) ≤15 mmHg. All PH patients were diagnosed by right heart catheterization according to the ESC/ERS Guidelines for the Diagnosis and Treatment of Pulmonary Hypertension available in enrollment period (19–21). Individuals without PH or chronic lung disease were included as controls. The study protocol was approved by the Ethics Committee of Shanghai Pulmonary Hospital, and written informed consent was obtained from all participants.

### Data collection

Demographic data, including age and gender, were collected from all participants. For PH patients, body mass index (BMI), 6-minute walking distance (6MWD), and World Health Organization functional class (WHO-FC) were assessed. Venous blood samples were collected for laboratory examinations, including interferon-α (IFN-α), interferon-γ (IFN-γ), N-terminal pro-brain natriuretic peptide (NT-proBNP), uric acid (UA), C-reactive protein (CRP), and lactic acid (LA). Hemodynamic parameters obtained by right heart catheterization included mean right atrial pressure (mRAP), mean pulmonary arterial wedge pressure (mPAWP), mPAP, PVR, cardiac output (CO), cardiac index (CI), and pulmonary arterial oxygen saturation (PA-SaO_2_). The primary endpoint of this study was all-cause mortality.

### Assessment of peripheral blood immunophenotype and plasma IFNs concentration

Peripheral blood samples (10 ml) were collected from all participants via the femoral vein and stored in both anticoagulant and procoagulant tubes. The samples were used to assess peripheral blood immunophenotypes and to isolate plasma for further analysis. Plasma concentrations of IFNs were measured using a Luminex-based immunoassay. Human IFN-α ProcartaPlex Simplex (EPX01A-10216-901, Thermo Fisher Scientific) and Human IFN-γ ProcartaPlex Simplex (EPX01A-10228-901, Thermo Fisher Scientific) kits were used following the manufacturer's instructions.

Briefly, capture beads were added to 96-well plates, followed by the addition of samples and standards. Plates were sealed and incubated for 2 h at room temperature (RT), then washed twice using a handheld magnetic plate washer (EPX-55555-000, Thermo Fisher Scientific). Detection antibodies were added and incubated for 30 min at RT, followed by additional washing. Streptavidin-PE was then added and incubated for 30 min at RT. After a final wash, the beads were resuspended, and data were acquired using a Luminex 200 system (Thermo Fisher Scientific).

### Statistical analysis

The Shapiro–Wilk test was used to assess the normality of continuous variables. Normally distributed data were presented as mean ± SD, while non-normally distributed data were presented as median (25th–75th percentile). Differences between groups were analyzed using unpaired Student's *t*-tests with Welch correction or one-way ANOVA with *post hoc* Tukey's tests. Categorical variables were presented as frequencies (percentages) and compared using the Pearson *χ*^2^ test.

Logistic regression models were applied to evaluate the association between IFN levels and PH risk. Receiver operating characteristic (ROC) analysis was used to determine optimal cutoff values. Correlations between variables were assessed using Spearman's rank correlation coefficient (*r*_s_). Cox proportional hazards regression was performed to analyze the relationship between variables and patient outcomes, while Kaplan–Meier survival curves were used to evaluate survival differences according to IFN-α levels. The predictive performance of prognostic models was assessed using ROC analysis and decision curve analysis. A nomogram was constructed to estimate individual overall survival probabilities. A two-tailed *P*-value <0.05 was considered statistically significant. All statistical analyses were performed using SPSS version 30.0 (IBM Corp.) and RStudio version 4.4.2.

## Results

### Baseline characteristics

The detailed baseline characteristics of the study population are summarized in Table 1, including 875 PH patients (mean age: 53.1 ± 17.2 years; males: 36.1%) and 182 age- and gender-matched controls (mean age: 51.0 ± 14.5 years; males: 42.3%). PH patients were classified into 4 subgroups (G1/G2/G3/G4: 448/116/236/75, respectively), and 161 patients reached the primary endpoint during follow-up. PAH patients were further classified into IPAH, CHD and CTD (180/88/180, respectively). The mean 6MWD among PH patients was 345.8 ± 134.8 meters. The median NT-proBNP concentration was 1,680.7 pg/mL (interquartile range: 218.0–1,983.5 pg/mL). Most patients were classified into WHO functional class II (*n* = 284, 32.5%) or III (*n* = 508, 58.1%).

**Table 1:** Baseline characteristics in patients with PH and controls.

	PH (*n* = 875)	Controls (*n* = 182)	*P* value
Age, years	53.1 ± 17.2	51.0 ± 14.5	0.090
Male, *n* (%)	316 (36.1)	77 (42.3)	0.116
BMI, kg/m^2^	22.4 ± 4.1	—	—
Classification, n			
G1/G2/G3/G4	448/116/236/75	—	—
IPAH/CHD/CTD	180/88/180	—	—
6MWD, m	345.8 ± 134.8	—	—
NT-proBNP, pg/mL	1,680.7 (218.0, 1,983.5)		—
UA, μmol/L	370.0 (293.0, 461.5)	—	—
CRP, mg/L	3.2 (3.1, 6.7)	—	—
LA, mmol/L	1.6 (1.2, 2.1)	—	
IFN-α, pg/mL	2.4 (1.4, 3.4)	1.6 (0.2, 2.7)	<0.001
IFN-γ, pg/mL	2.1 (1.2, 3.1)	1.5 (0.6, 2.6)	<0.001
WHO FC, I/II/III/IV, n	23/284/508/60		
Hemodynamics			
mRAP, mm Hg	4 (1, 7)	—	—
mPAP, mm Hg	43.0 (32.0, 54.0)	—	—
mPAWP, mm Hg	8 (5, 12)	—	—
PVR, Wood units	7.4 (4.0, 11.9)	—	—
CO, L/min	4.8 ± 1.5	—	—
CI, L/min/m^2^	3.0 ± 0.9	—	—
PA-SaO2, %	65.1 ± 8.6	—	—

Values are showed as means (±SD), medians (interquartile range), or *n* (%).

### Association of IFN-α and IFN-γ with PH risk

**Figure 1:**
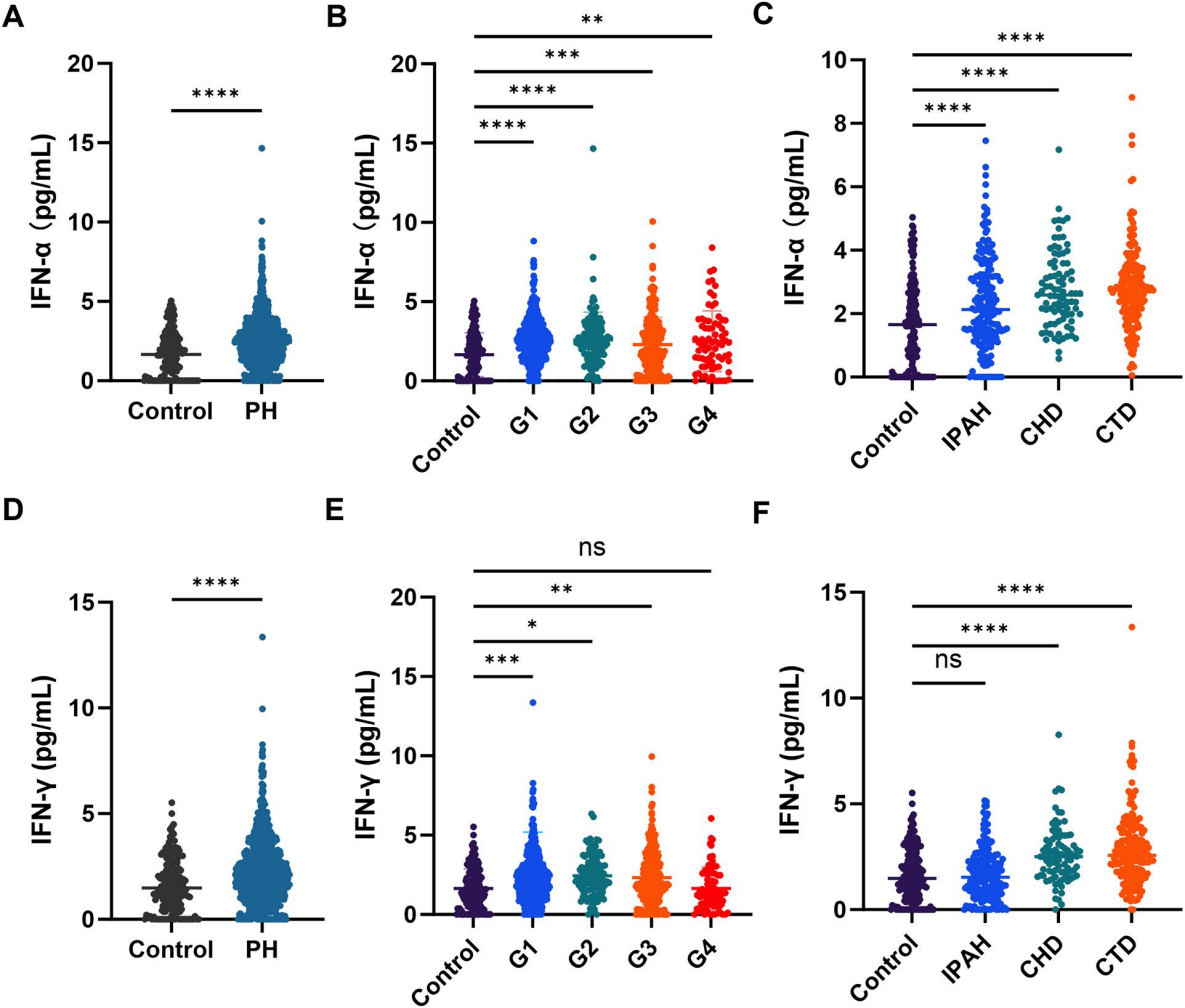
Plasma IFNs levels in patients with PH. **(A)** Plasma IFN-α levels between controls and PH patients (*n* = 182, *n* = 875); **(B)** plasma IFN-α levels between controls and PH subgroups (*n* = 182, *n* = 448, *n* = 116, *n* = 236, *n* = 75); **(C)** plasma IFN-α levels between controls and PAH subtypes (*n* = 182, *n* = 180, *n* = 88, *n* = 180); **(D)** plasma IFN-γ levels between controls and PH patients (*n* = 182, *n* = 875); **(E)** plasma IFN-γ levels between controls and PH subgroups (*n* = 182, *n* = 448, *n* = 116, *n* = 236, *n* = 75); **(F)** plasma IFN-γ levels between controls and PAH subtypes (*n* = 182, *n* = 180, *n* = 88, *n* = 180).

To examine the relationship between interferon levels and PH, plasma concentrations of IFN-α and IFN-γ were compared between PH patients and controls. Both plasma IFN-α and IFN-γ levels were significantly elevated in PH patients (Figures 1A,D). IFN-α levels were markedly increased across all four PH subgroups and all three PAH subtypes (Figures 1B,C). In contrast, IFN-γ levels were elevated only in G1–G3 PH and in CHD-PAH and CTD-PAH subtypes (Figures 1E,F).

**Figure 2:**
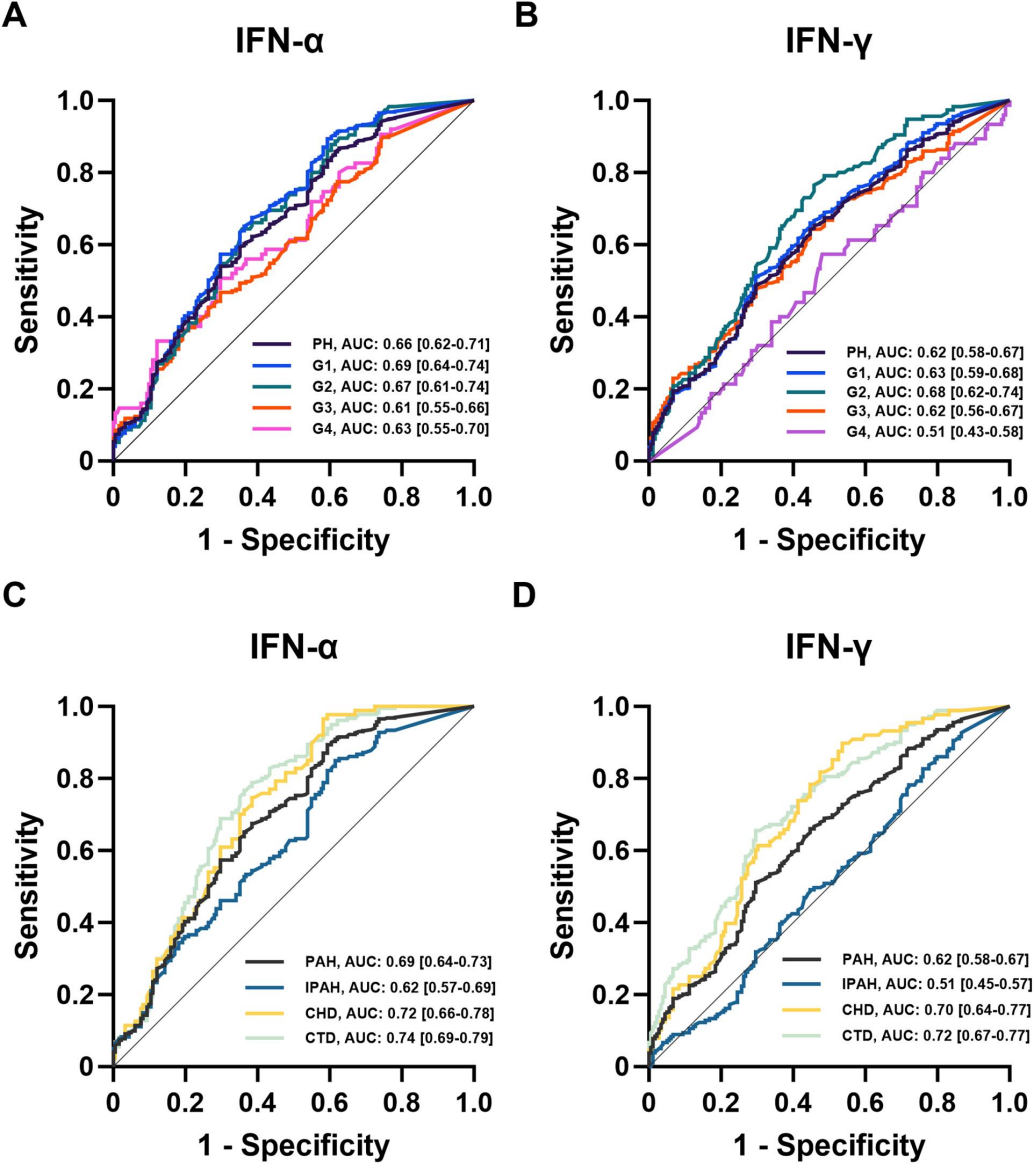
ROC analysis of IFNs in patients with PH. **(A,B)** ROC analysis of plasma IFNs in controls and PH subgroups (*n* = 182, *n* = 875, *n* = 448, *n* = 116, *n* = 236, *n* = 75); **(C,D)** ROC analysis of plasma IFNs in PAH subtypes (*n* = 448, *n* = 180, *n* = 88, *n* = 180).

Logistic regression and receiver operating characteristic (ROC) analyses were performed to evaluate the association between IFN levels and PH risk (Table 2, Figure 2). Both IFN-α and IFN-γ were associated with increased disease risk, with IFN-α showing stronger predictive performance, particularly in patients with G4-PH and IPAH. The area under the curve (AUC) values for IFN-α among G1-PH, G2-PH, G3-PH, and G4-PH were 0.66 (sensitivity 54%, specificity 70%, *P* < 0.0001), 0.69 (sensitivity 89%, specificity 41%, *P* < 0.0001), 0.67 (sensitivity 64%, specificity 64%, *P* < 0.0001), and 0.61 (sensitivity 47%, specificity 70%, *P* < 0.001), respectively (Figure 2A). In comparison, AUC values for IFN-γ were 0.62 (sensitivity 64%, specificity 55%, *P* < 0.0001), 0.63 (sensitivity 51%, specificity 70%, *P* < 0.0001), 0.68 (sensitivity 77%, specificity 54%, *P* < 0.0001), 0.62 (sensitivity 64%, specificity 55%, *P* < 0.0001), and 0.51 (sensitivity 57%, specificity 52%, *P* > 0.05) when compared with controls (Figure 2B). Notably, within the PAH subgroup, IFN-α demonstrated superior predictive ability compared with IFN-γ, particularly in CTD-PAH patients (Figures 2C,D).

**Table 2:** Logistic regression of IFNs to predict risk of disease in patients with PH and controls.

Dependent	Independent (pg/mL)	OR (95% CI)			
		Model 1	*P* value	Model 2	*P* value
PH	IFN-α	1.541 (1.358–1.750)	<0.001	1.544 (1.360–1.753)	<0.001
	IFN-γ	1.407 (1.236–1.602)	<0.001	1.408 (1.236–1.04)	<0.001
G1-PH	IFN-α	1.745 (1.505–2.023)	<0.001	1.805 (1.546–2.107)	<0.001
	IFN-γ	1.456 (1.262–1.680)	<0.001	1.490 (1.283–1.730)	<0.001
G2-PH	IFN-α	1.545 (1.289–1.851)	<0.001	1.700 (1.372–2.107)	<0.001
	IFN-γ	1.661 (1.360–2.030)	<0.001	1.802 (1.418–2.291)	<0.001
G3-PH	IFN-α	1.298 (1.138–1.482)	<0.001	1.280 (1.110–1.476)	<0.001
	IFN-γ	1.374 (1.191–1.586)	<0.001	1.325 (1.136–1.546)	<0.001
G4-PH	IFN-α	1.394 (1.172–1.658)	<0.001	1.391 (1.152–1.681)	<0.001
	IFN-γ	1.008 (0.815–1.246)	0.942	1.016 (0.810–1.275)	0.890
IPAH	IFN-α	1.400 (1.203–1.628)	<0.001	1.457 (1.238–1.714)	<0.001
	IFN-γ	1.037 (0.876–1.228)	0.672	1.008 (0.842–1.207)	0.930
CHD	IFN-α	1.823 (1.476–2.252)	<0.001	1.898 (1.513–2.381)	<0.001
	IFN-γ	1.767 (1.417–2.204)	<0.001	1.783 (1.412–2.252)	<0.001
CTD	IFN-α	1.993 (1.650–2.408)	<0.001	1.984 (1.617–2.434)	<0.001
	IFN-γ	1.834 (1.533–2.195)	<0.001	1.968 (1.592–2.433)	<0.001

Model 1, no adjustment; Model 2, adjusted for age and gender; OR, Odds ratio.

### Association of IFN-α with survival in PH

**Figure 3:**
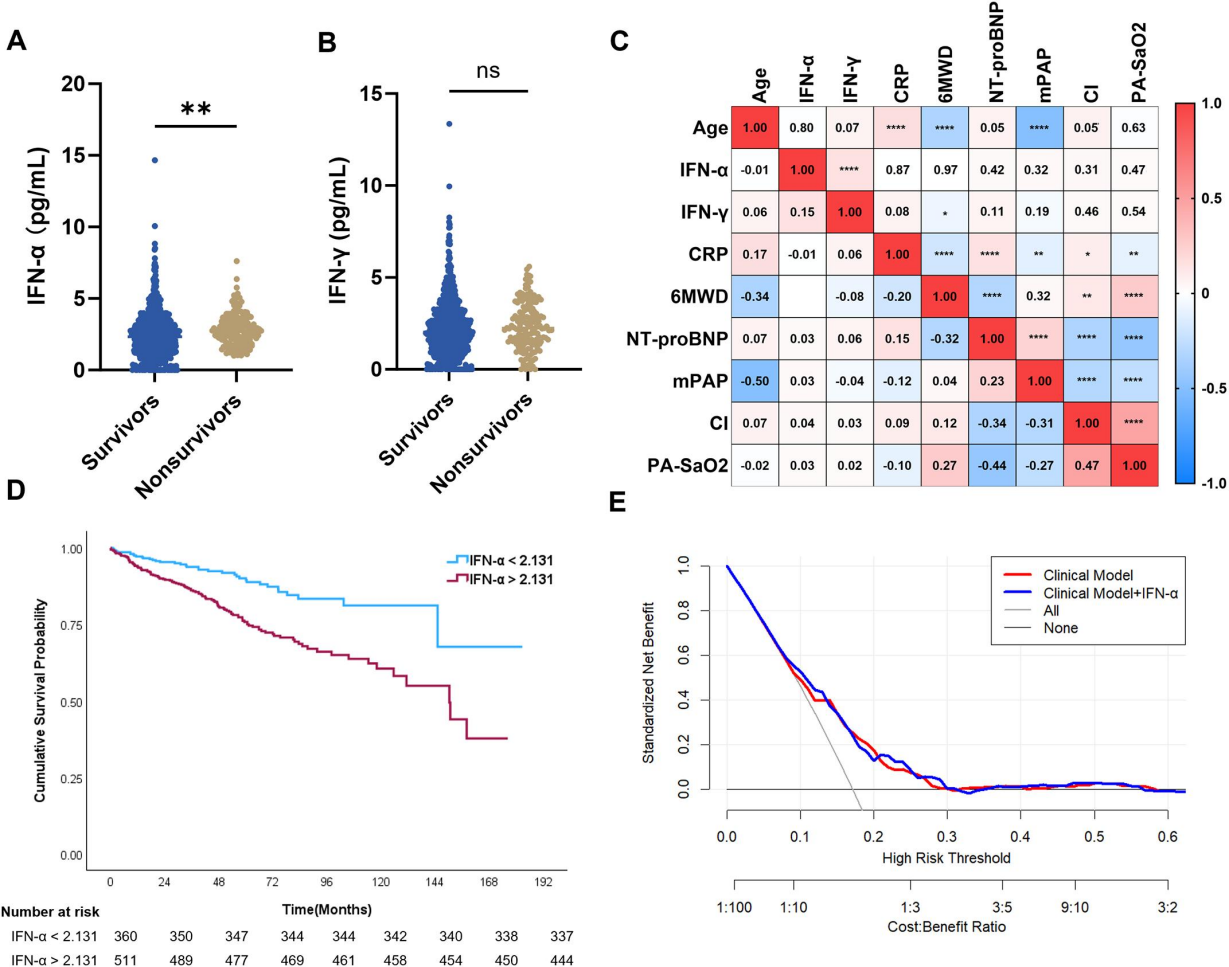
Associations of plasma IFN-α with survival of patients. **(A,B)** Plasma IFN-α levels between survivors and nonsurvivors of PH patients (*n* = 161, *n* = 714); **(C)** Spearman correlation analysis of plasma IFNs with clinical parameters in patients with PH; **(D)** Kaplan–Meier survival curve in PH patients with high/low levels of IFN-α; **(E)** decision curve analysis of Cox regression models, clinical model includes WHO-FC, 6MWD and NT-proBNP.

During follow-up, 161 PH patients died, including 87 with G1-PH, 18 with G2-PH, and 56 with G3-PH. Among PAH patients, 44 IPAH, 18 CHD-PAH, and 25 CTD-PAH patients did not survive. Plasma IFN-α levels were significantly higher in nonsurvivors compared with survivors (*P* < 0.01; Figure 3A), whereas IFN-γ levels were not significantly different (*P* = 0.34; Figure 3B).

Cox regression and Kaplan–Meier survival analyses were conducted to evaluate the relationship between IFN levels and overall survival. Univariate Cox regression revealed that IFN-α levels were significantly associated with mortality in PH patients (HR: 1.122, 95% CI: 1.032–1.219, *P* = 0.007), while IFN-γ was not (HR: 1.087, 95% CI: 0.990–1.193, *P* = 0.081; Table 3). Pearson's correlation analysis showed that IFN-α levels were modestly correlated with IFN-γ (*r*_s_ = 0.15, *P* < 0.0001) but not with CRP (*P* = 0.87; Figure 3C), another classical inflammatory marker.

**Table 3:** Cox regression of IFNs to predict survival rate of PH patients.

	HR	95% CI	*P* value
Univariate Cox regression			
Age, per year	1.019	1.009–1.029	<0.001
Gender, female	0.501	0.368–0.684	<0.001
WHO-FC, per class	2.127	1.651–2.740	<0.001
IFN-α, per pg/mL	1.122	1.032–1.219	0.007
IFN-γ, per pg/mL	1.087	0.990–1.193	0.081
CRP, per mg/L	1.011	1.002–1.020	0.019
6MWD, <345.8 m	0.423	0.287–0.623	<0.001
NT-proBNP, per quartile	2.862	2.019–4.056	<0.001
mPAP, per mm Hg	0.999	0.990–1.008	0.842
CI, per L/min/m^2^	0.824	0.681–0.996	0.045
PA-SaO2, per %	0.955	0.939–0.972	<0.001
Multivariate Cox regression			
Gender, female	0.549	0.375–0.805	0.002
WHO-FC, per class	1.407	1.009–1.963	0.044
6MWD, <345.8 m	0.576	0.383–0.868	0.008
NT-proBNP, per quartile	1.709	1.398–2.089	<0.001
IFN-α, per pg/mL	1.120	1.001–1.253	0.048

In multivariate Cox regression, IFN-α remained a significant independent predictor of mortality (HR: 1.120, 95% CI: 1.001–1.253, *P* = 0.048), together with gender (HR: 0.549, 95% CI: 0.375–0.805, *P* = 0.002), WHO-FC (HR: 1.407, 95% CI: 1.009–1.963, *P* = 0.044), 6MWD (HR: 0.576, 95% CI: 0.383–0.868, *P* = 0.008), and NT-proBNP (HR:1.709, 95% CI: 1.398–2.089, *P* < 0.001; Table 3). However, age had no significance in the multivariate Cox regression model (HR: 1.007, 95% CI: 0.995–1.020, *P* = 0.247). ROC analysis between survivors and nonsurvivors identified an optimal IFN-α cutoff value of 2.131 pg/mL (AUC: 0.61, 95% CI: 0.56–0.65, *P* < 0.0001). Kaplan–Meier analysis demonstrated that patients with IFN-α >2.131 pg/mL had significantly lower overall survival (Figure 3D).

To evaluate the prognostic performance of multivariate Cox regression model, we compared it with a clinical model that only included clinical variables (WHO-FC, 6MWD and NT-proBNP). Decision curve analysis indicated that both models provided positive clinical net benefits for intervention thresholds between 8% and 30%, with the Cox regression model offering additional net benefit within the 20%–30% high-risk range (Figure 3E).

**Figure 4:**
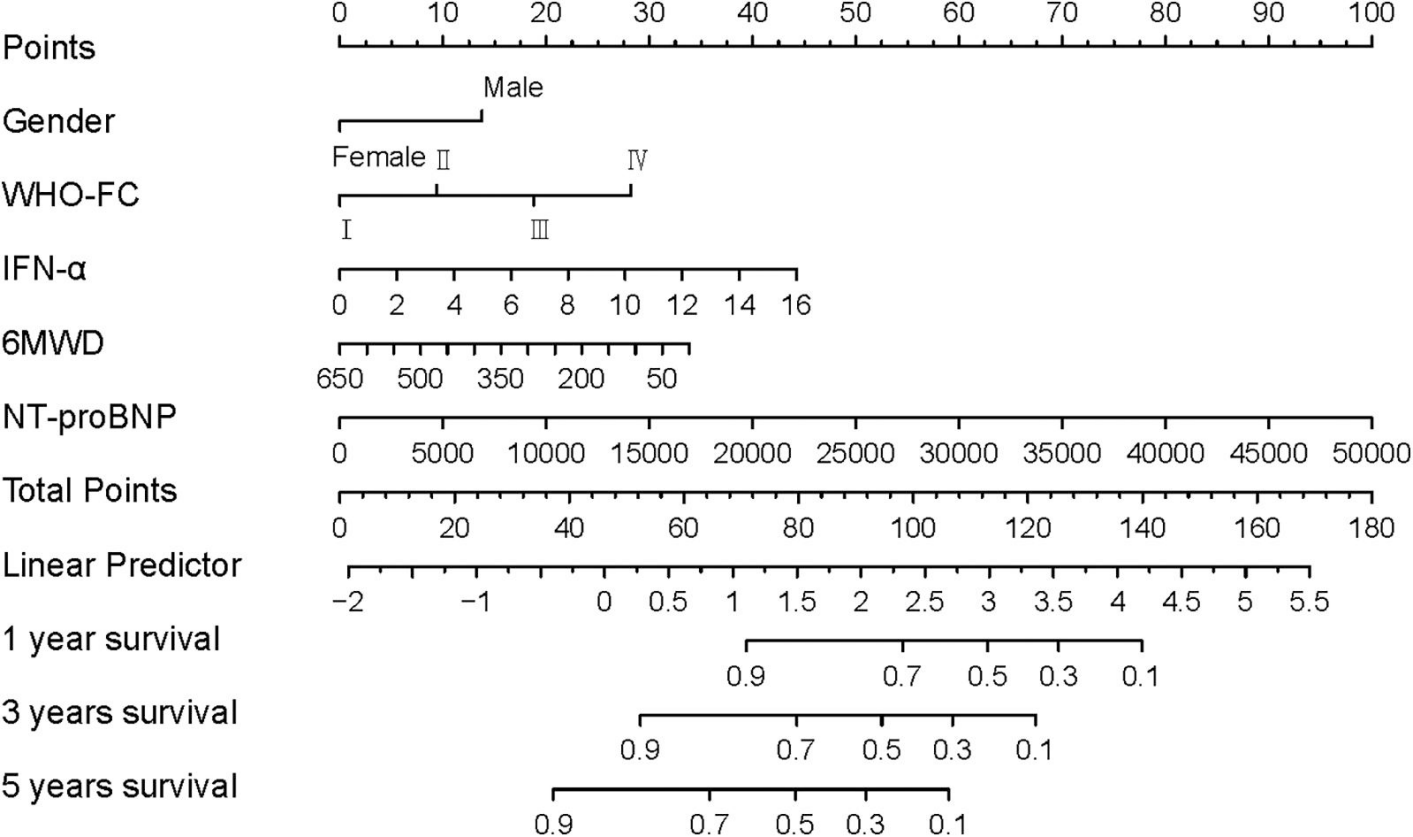
Nomogram for survival prediction in PH patients.

Finally, a prognostic nomogram incorporating gender, WHO-FC, 6MWD, NT-proBNP, and IFN-α was constructed to predict individual overall survival (Figure 4). In the nomogram, female patients were assigned a score of 0, while male patients were assigned 13 points. WHO-FC contributed 0–28 points (from class I to IV), 6MWD contributed 0–33 points (decreasing with distance), and IFN-α contributed 0–44 points (increasing with higher concentrations).

## Discussion

In this study, we compared plasma IFN levels between patients with PH and age- and gender-matched controls, and further evaluated the predictive and prognostic significance of IFNs in PH. Both IFN-α and IFN-γ levels were elevated in PH patients, suggesting a systemic inflammatory state associated with disease development. Notably, IFN levels varied among different PH groups and PAH subtypes, implying heterogeneous immune activation across distinct pathophysiological mechanisms. Logistic regression analysis demonstrated that IFN-α was a more sensitive predictor of PH risk than IFN-γ, and its elevation was independently associated with poorer survival outcomes. These findings collectively suggest that IFN-α serves as both a biomarker for PH susceptibility and an indicator of disease prognosis.

The correlation between IFN-α and IFN-γ observed in this study indicates a shared activation of inflammatory pathways, whereas their independence from conventional inflammatory markers such as NT-proBNP and CRP supports their unique contribution to PH pathobiology. In multivariate Cox regression analysis, IFN-α remained an independent prognostic factor, while CRP lost significance, underscoring the potential specificity of IFN-α as a marker of disease progression rather than a general indicator of systemic inflammation.

Previous studies have established the involvement of multiple cytokines, including IL-6, IL-8, and IL-10, in the pathogenesis and progression of PH (12, 22, 23). However, the role of interferons in PH has been less well defined. Our study is the first to demonstrate that elevated IFNs, particularly IFN-α, are associated not only with PH risk but also with long-term prognosis. Furthermore, our analysis was based on a relatively large cohort (875 PH patients and 182 matched controls), enhancing the robustness of these findings. Interestingly, subgroup analysis revealed that IFNs achieved higher discriminative power (AUC > 0.70) in PH patients with CHD-PAH and CTD-PAH, suggesting that IFN-mediated inflammation may be especially relevant in these etiological subtypes.

The prognostic model established in this study provides translational insights for PH management. Patients with elevated IFN-α exhibited significantly reduced overall survival. In our nomogram, IFN-α performed comparably to established clinical predictors such as WHO-FC and 6MWD. Given the fact that NT-proBNP often shows wide interindividual variability, IFN-α could be a useful complement to existing prognostic tools and may aid in clinical risk stratification.

Interferons are pleiotropic cytokines with dual roles in host defense and immune regulation. While they are therapeutically applied in diseases such as multiple sclerosis and viral hepatitis (24–26), several reports have described interferon-induced PAH as an adverse effect (27–29). Experimental evidence has implicated monocyte/macrophage activation and vascular smooth muscle cell proliferation in interferon-mediated vascular remodeling (30). Our findings align with these observations, suggesting that sustained IFN signaling may contribute to pulmonary vascular pathology. These insights emphasize the need for caution when using IFN-based therapies, especially in individuals with pre-existing risk factors for PH (28, 31).

This study has several limitations. Firstly, this study was conducted at a single center, which might limit the generalizability of the findings and introduce selection bias. Secondly, although we identified significant associations between IFN levels and PH risk, potential confounders such as BMI were not included in the logistic models, and mechanistic pathways linking IFNs to PH progression remain to be elucidated. Additionally, control-group data for 6MWD and NT-proBNP were unavailable, preventing direct comparison of diagnostic accuracy with IFNs. The ROC analysis also showed that IFNs alone had modest diagnostic value (AUC < 0.8), indicating limited clinical utility as standalone biomarkers. Although ROC analysis identified an IFN-α cutoff of 2.131 pg/mL to predict survival in our cohort, the discriminative performance was modest (AUC = 0.61). Previously published cutoffs vary widely (e.g., ∼0.6 pg/mL, 14.7 pg/mL and >100 pg/mL in different disease contexts and cohorts), reflecting differences in patient populations and assay platforms (32–34). Nevertheless, incorporation of IFN-α into a prognostic model yielded meaningful incremental benefit, underscoring its translational relevance. Finally, we lacked detailed prognostic information such as rehospitalization or death caused by cardiovascular or not-cardiovascular events, which limits the prognostic value of our model. Meanwhile, the lack of comprehensive echocardiographic, pulmonary function and other clinical data may have an impact on the prognostic outcome of PH patients. Future multicenter prospective studies with larger cohorts and long-term follow up are warranted to validate these findings.

In conclusion, by quantifying plasma IFN-α levels and correlating them with disease severity and survival, our study identified IFN-α as a novel and independent biomarker for PH risk and prognosis. These findings highlighted the potential clinical value of incorporating IFN-α into prognostic assessment and raised the possibility that modulation of IFN signaling could represent a future therapeutic avenue in pulmonary hypertension.

## Data Availability

The raw data supporting the conclusions of this article will be available from the corresponding author upon reasonable request.

## References

[1] Mocumbi A Humbert M Saxena A Jing Z-C Sliwa K Thienemann F Pulmonary hypertension. Nat Rev Dis Primers. (2024) 10(1):1. 10.1038/s41572-023-00486-7 38177157

[2] Poch D Mandel J. Pulmonary hypertension. Ann Intern Med. (2021) 174(4):Itc49–64. 10.7326/AITC202104200 33844574

[3] Simonneau G Gatzoulis MA Adatia I Celermajer D Denton C Ghofrani A Updated clinical classification of pulmonary hypertension. J Am Coll Cardiol. (2013) 62(25 Suppl):D34–41. 10.1016/j.jacc.2013.10.029 24355639

[4] Ruopp NF Cockrill BA. Diagnosis and treatment of pulmonary arterial hypertension: a review. Jama. (2022) 327(14):1379–91. 10.1001/jama.2022.4402 35412560

[5] Platanias LC. Mechanisms of type-I- and type-II-interferon-mediated signalling. Nat Rev Immunol. (2005) 5(5):375–86. 10.1038/nri1604 15864272

[6] Thompson AAR Lawrie A. Targeting vascular remodeling to treat pulmonary arterial hypertension. Trends Mol Med. (2017) 23(1):31–45. 10.1016/j.molmed.2016.11.005 27989641

[7] Ye Y Xu Q Wuren T. Inflammation and immunity in the pathogenesis of hypoxic pulmonary hypertension. Front Immunol. (2023) 14:1162556. 10.3389/fimmu.2023.1162556 37215139 PMC10196112

[8] Zhao H Song J Li X Xia Z Wang Q Fu J The role of immune cells and inflammation in pulmonary hypertension: mechanisms and implications. Front Immunol. (2024) 15:1374506. 10.3389/fimmu.2024.1374506 38529271 PMC10962924

[9] Rabinovitch M Guignabert C Humbert M Nicolls MR. Inflammation and immunity in the pathogenesis of pulmonary arterial hypertension. Circ Res. (2014) 115(1):165–75. 10.1161/CIRCRESAHA.113.301141 24951765 PMC4097142

[10] Wang R-R Yuan T-Y Wang J-M Chen Y-C Zhao J-L Li M-T Immunity and inflammation in pulmonary arterial hypertension: from pathophysiology mechanisms to treatment perspective. Pharmacol Res. (2022) 180:106238. 10.1016/j.phrs.2022.106238 35504356

[11] Gairhe S Awad KS Dougherty EJ Ferreyra GA Wang S Yu Z-X Type I interferon activation and endothelial dysfunction in caveolin-1 insufficiency-associated pulmonary arterial hypertension. Proc Natl Acad Sci U S A. (2021) 118(11):e2010206118. 10.1073/pnas.2010206118 33836561 PMC7980434

[12] Soon E Holmes AM Treacy CM Doughty NJ Southgate L Machado RD Elevated levels of inflammatory cytokines predict survival in idiopathic and familial pulmonary arterial hypertension. Circulation. (2010) 122(9):920–7. 10.1161/CIRCULATIONAHA.109.933762 20713898

[13] Agrawal V Kropski JA Gokey JJ Kobeck E Murphy MB Murray KT Myeloid cell derived IL1β contributes to pulmonary hypertension in HFpEF. Circ Res. (2023) 133(11):885–98. 10.1161/CIRCRESAHA.123.323119 37929582 PMC10655859

[14] Hirsch K Nolley S Ralph DD Zheng Y Altemeier WA Rhodes CJ Circulating markers of inflammation and angiogenesis and clinical outcomes across subtypes of pulmonary arterial hypertension. J Heart Lung Transplant. (2023) 42(2):173–82. 10.1016/j.healun.2022.10.026 36470771 PMC9840657

[15] Kumar S Mickael C Kumar R Prasad RR Campbell NV Zhang H Single cell transcriptomic analyses reveal diverse and dynamic changes of distinct populations of lung interstitial macrophages in hypoxia-induced pulmonary hypertension. Front Immunol. (2024) 15:1372959. 10.3389/fimmu.2024.1372959 38690277 PMC11059952

[16] Thenappan T Ormiston ML Ryan JJ Archer SL. Pulmonary arterial hypertension: pathogenesis and clinical management. Br Med J. (2018) 360:j5492. 10.1136/bmj.j5492 29540357 PMC6889979

[17] Deng Y Guo S-l Li J-q Xie S-s Zhou Y-c Wei B Interferon regulatory factor 7 inhibits rat vascular smooth muscle cell proliferation and inflammation in monocrotaline-induced pulmonary hypertension. Life Sci. (2021) 264:118709. 10.1016/j.lfs.2020.118709 33152351

[18] Ruffenach G Medzikovic L Aryan L Sun W Lertpanit L O’Connor E Intestinal IFN*α*4 promotes 15-HETE diet-induced pulmonary hypertension. Respir Res. (2024) 25(1):419. 10.1186/s12931-024-03046-z 39609844 PMC11606228

[19] Humbert M Kovacs G Hoeper MM Badagliacca R Berger RMF Brida M 2022 ESC/ERS guidelines for the diagnosis and treatment of pulmonary hypertension. Eur Heart J. (2022) 43(38):3618–731. 10.1093/eurheartj/ehac237 36017548

[20] Galiè N Hoeper MM Humbert M Torbicki A Vachiery JL Barbera JA Guidelines for the diagnosis and treatment of pulmonary hypertension. Eur Respir J. (2009) 34(6):1219–63. 10.1183/09031936.00139009 19749199

[21] Galiè N Humbert M Vachiery J-L Gibbs S Lang I Torbicki A 2015 ESC/ERS guidelines for the diagnosis and treatment of pulmonary hypertension: The Joint Task Force for the Diagnosis and Treatment of Pulmonary Hypertension of the European Society of Cardiology (ESC) and the European Respiratory Society (ERS): Endorsed by: Association for European Paediatric and Congenital Cardiology (AEPC), International Society for Heart and Lung Transplantation (ISHLT). Eur Heart J. (2016) 37(1):67–119. 10.1093/eurheartj/ehv317 26320113

[22] Huertas A Tu L Humbert M Guignabert C. Chronic inflammation within the vascular wall in pulmonary arterial hypertension: more than a spectator. Cardiovasc Res. (2020) 116(5):885–93. 10.1093/cvr/cvz308 31813986

[23] Liu S-F Nambiar Veetil N Li Q Kucherenko MM Knosalla C Kuebler WM. Pulmonary hypertension: linking inflammation and pulmonary arterial stiffening. Front Immunol. (2022) 13:959209. 10.3389/fimmu.2022.959209 36275740 PMC9579293

[24] Chevaliez S Pawlotsky JM. Interferon-based therapy of hepatitis C. Adv Drug Deliv Rev. (2007) 59(12):1222–41. 10.1016/j.addr.2007.07.002 17869375

[25] Conway D Cohen JA. Combination therapy in multiple sclerosis. Lancet Neurol. (2010) 9(3):299–308. 10.1016/S1474-4422(10)70007-7 20170843

[26] Goldstein D Laszlo J. The role of interferon in cancer therapy: a current perspective. CA Cancer J Clin. (1988) 38(5):258–77. 10.3322/canjclin.38.5.258 2458171

[27] Montani D Seferian A Savale L Simonneau G Humbert M. Drug-induced pulmonary arterial hypertension: a recent outbreak. Eur Respir Rev. (2013) 22(129):244–50. 10.1183/09059180.00003313 23997051 PMC9487364

[28] Savale L Sattler C Günther S Montani D Chaumais M-C Perrin S Pulmonary arterial hypertension in patients treated with interferon. Eur Respir J. (2014) 44(6):1627–34. 10.1183/09031936.00057914 25323231

[29] Seferian A Chaumais M-C Savale L Günther S Tubert-Bitter P Humbert M Drugs induced pulmonary arterial hypertension. Presse Med. (2013) 42(9 Pt 2):e303–10. 10.1016/j.lpm.2013.07.005 23972547

[30] Schermuly RT Ghofrani HA Wilkins MR Grimminger F. Mechanisms of disease: pulmonary arterial hypertension. Nat Rev Cardiol. (2011) 8(8):443–55. 10.1038/nrcardio.2011.87 21691314 PMC7097518

[31] L’Heureux M Swiatek K Guthrie R Brath L Grinnan D. Pulmonary hypertension: advances in risk assessment and emerging procedures. Am J Respir Crit Care Med. (2020) 202(5):745–7. 10.1164/rccm.201912-2484RR 32525396

[32] Fayed A El Menyawi MM Ghanema M Shaker O Elgohary R. Measurement of serum interferon alpha in Egyptian patients with systemic lupus erythematosus and evaluation of its effect on disease activity: a case-control study. Reumatismo. (2020) 72(3):145–53. 10.4081/reumatismo.2020.1308 33213127

[33] Nagaoka K Kawasuji H Takegoshi Y Murai Y Kaneda M Kimoto K Predictive values of immune indicators on respiratory failure in the early phase of COVID-19 due to delta and precedent variants. Front Immunol. (2023) 14:1197436. 10.3389/fimmu.2023.1197436 37731495 PMC10507327

[34] Whittall Garcia LP Gladman DD Urowitz M Bonilla D Schneider R Touma Z Interferon-α as a biomarker to predict renal outcomes in lupus nephritis. Lupus Sci Med. (2024) 11(2):e001347. 10.1136/lupus-2024-001347 39613334 PMC11605838

